# Effect of phage vB_EcoM_FJ1 on the reduction of ETEC O9:H9 infection in a neonatal pig cell line

**DOI:** 10.1186/s13567-023-01157-x

**Published:** 2023-03-22

**Authors:** Alice Ferreira, Daniela Silva, Carina Almeida, Maria Elisa Rodrigues, Sónia Silva, Joana Castro, Dalila Mil-Homens, Isidro García-Meniño, Azucena Mora, Mariana Henriques, Ana Oliveira

**Affiliations:** 1 ALS ControlVet, Zona Industrial de Tondela ZIMII, Lote 6, 3460-605 Tondela, Portugal; 2 CEB - Centre of Biological Engineering, University of Minho, 4710-057 Braga, Portugal; 3 LABBELS- Associate Laboratory, 4800-122 Guimarães, Portugal; 4 I.P - National Institute for Agrarian and Veterinarian Research (INIAV), Rua Dos Lagidos, 4485-655 Vila Do Conde, Portugal; 5 Institute for Bioengineering and Biosciences (IBB), Instituto Superior Técnico, 1049-001 Lisbon, Portugal; 6 Laboratorio de Referencia de Escherichia Coli (LREC), Departamento de Microbioloxía E Parasitoloxía, Facultade de Veterinaria, Universidade de Santiago de Compostela (USC), 27002 Lugo, Spain; 7 Instituto de Investigación Sanitaria de Santiago de Compostela (IDIS), 15706 Santiago de Compostela, Spain; 8 Department for Biological Safety, German Federal Institute for Risk Assessment, Berlin, Germany

**Keywords:** Swine colibacillosis, ETEC, bacteriophage, pig neonatal cell line, BIMs

## Abstract

Enterotoxigenic *Escherichia coli* (ETEC) colonizes the intestine of young pigs causing severe diarrhoea and consequently bringing high production costs. The rise of antibiotic selective pressure together with ongoing limitations on their use, demands new strategies to tackle this pathology. The pertinence of using bacteriophages as an alternative is being explored, and in this work, the efficacy of phage vB_EcoM_FJ1 (FJ1) in reducing the load of ETEC EC43-Ph (serotype O9:H9 expressing the enterotoxin STa and two adhesins F5 and F41) was assessed. Foreseeing the oral application on piglets, FJ1 was encapsulated on calcium carbonate and alginate microparticles, thus preventing phage release under adverse conditions of the simulated gastric fluid (pH 3.0) and allowing phage availability in simulated intestinal fluid (pH 6.5). A single dose of encapsulated FJ1, provided to IPEC-1 cultured cells (from intestinal epithelium of piglets) previously infected by EC43, provided bacterial reductions of about 99.9% after 6 h. Although bacteriophage-insensitive mutants (BIMs) have emerged from treatment, the consequent fitness costs associated with this new phenotype were demonstrated, comparatively to the originating strain. The higher competence of the pig complement system to decrease BIMs’ viability, the lower level of colonization of IPEC-1 cells observed with these mutants, and the increased survival rates and health index recorded in infected *Galleria mellonella* larvae supported this observation. Most of all, FJ1 established a proof-of-concept of the efficiency of phages to fight against ETEC in piglet intestinal cells.

**Supplementary Information:**

The online version contains supplementary material available at 10.1186/s13567-023-01157-x.

## Introduction

*Escherichia coli* is a gram-negative bacterium that is naturally part of the intestinal microbiota. However, certain pathogenic strains of *E. coli* are associated with pathologies in the animal production industry, as is the case of swine enteric colibacillosis [1]. This infection occurs in porcine farms, and frequently affects nursing and weaning pigs [2]. Outbreaks are mainly associated with enterotoxigenic *E. coli* (ETEC) and require the implementation of expensive measures to control the disease, such as antibiotic treatments, vaccination, or feed supplements [2, 3]. This pathotype is characterized by the presence of surface proteins (F4, F5, F6, F18, and F41) that recognize and bind to the small intestine enterocytes and also by the production of different types of toxins: heat-stable (STa and STb) and heat-labile (LT) enterotoxins [2]. The enteroaggregative heat-stable toxin EAST-1 also plays a role in pigs with post-weaning diarrhoea; however, its mechanism has not been fully elucidated [2, 4]. These virulence factors are responsible for piglet dehydration, weight loss, and watery diarrhoea that can lead to animal death [2, 5].

It should be highlighted that the major structural components of the outer membrane of most gram-negative bacteria, such as ETEC, are the lipopolysaccharides (LPS), which are essential components of the cell envelope. Such structures also act as an effective permeability barrier against small, hydrophobic molecules, making bacteria innately resistant to many antimicrobial compounds. Furthermore, LPS, act as strong stimulators and modulators of innate immunity in eukaryotes. Briefly, these large glycolipid structures are composed of a polysaccharide region, the O antigen, anchored to the membrane by a core oligosaccharide, in turn, connected to the hydrophobic portion, lipid A. Specifically, the lipid A is highly immunostimulatory and the O antigen is the most diverse component of LPS. The variability of the O antigen structure and composition, sometimes, allows organisms to evade the host immune response by masking the more conserved core parts [6, 7].

Different alternatives to antibiotics are being used to prevent ETEC propagation in porcine farms. For example, the use of injectable vaccines in sows to elicit maternal immunity that protects piglets during the nursing period, or/and the administration of vaccines in the weaning period to provide mucosal immunity (e.g., oral delivery of fimbriae-containing vaccines, live attenuated vaccines, or vaccines with avirulent *E. coli* expressing fimbriae but lacking toxin production) [8]. Other products and strategies are being explored, such as feeding with antimicrobial minerals, organic acids, enzymes, functional feedstuff, prebiotic oligosaccharides, or selecting and breeding ETEC-resistant piglets [9, 10]. The new legislation on veterinary medicine [Regulation (EU) 2019/6] and medicated feed [Regulation (EU) 2019/4] came into force recently, limiting, among others, the use of antibiotic prophylaxis and metaphylaxis. The isolation of ETEC, carriers of plasmids conferring resistance to extended-spectrum beta-lactamases (ESBLs) and polymyxins is of great concern [3, 11, 12]. Such occurrences together with the limited efficacy of nowadays available antibiotics are pressing the investigation of new approaches, such as the use of bacteriophages (phages), to tackle ETEC infections [10]. These are strictly bacterial viruses, consisting of a protein capsid that involves the genetic material, most presenting a tail with tail fibers at the edge [13]. The use of phages for veterinary and human use has been proving to be safe [14, 15]. However, for therapy, only virulent phages (i.e., virions that replicate and cause bacterial lysis at the end of the infection [13, 16]) are acceptable. Ubiquitous in nature, phages are self-dosing and specific for a given host, avoiding dysbiosis [17, 18]. The emergence of bacteriophage-insensitive mutants (BIMs) has also been described. However, the developing resistance to phage may imply an associated environmental fitness cost to the host, compromising the performance of traits such as growth rates, virulence, or resistance to various antimicrobials [19]. On the other hand, something to consider in the case of planning the ingestion of phages is their high sensitivity to environments with low pH, as occurs in the stomach, known as an adverse environment for them [20, 21]. The encapsulation of viral particles using different approaches (e.g., liposomes, emulsion, whey protein, alginate) is being explored to mitigate these challenges [22]. Alginate is one of the most explored biomaterials for the production of capsules due to its lack of toxicity to cell tissues or immunogenicity [23]. For example, alginate-based microcapsules have already proved to protect phages from gastric conditions [20, 24, 25].

In this study, calcium carbonate and alginate (CaCO_3_/alginate) microcapsules containing phage vB_EcoM_FJ1 (FJ1) were characterized in gastrointestinal simulated fluids and its efficacy against colonized ETEC O9:H9 load in neonatal pig intestinal cultured cells was assessed. The virulence of BIMs was further evaluated in the pigs’ complement system, culture cells and *Galleria mellonella* model.

## Materials and methods

### Bacterial culture conditions and phage propagation

The ETEC strain EC43-Ph (EC43), previously serotyped as O9:H9, comprises the enterotoxin (STa) and two adhesins (F5 and F41). This diarrheagenic *E. coli* was recovered from a neonatal pig in a Spanish swine farm [3]. EC43 was cultivated in Lysogeny Broth (LB, NZYTech) or on LB agar (12 g L^−1^, VWR), at 37 °C. The phage vB_EcoM_FJ1 (FJ1) and its host HFJ1 were provided by the company ALS. Although FJ1 has been isolated from chickens’ litter, from an enrichment with the avian pathogenic *E. coli* strain HFJ1 (non-published data), this phage infects EC43, a swine-originating *E. coli* strain. FJ1 was then propagated on a mid-log grown host strain, HFJ1, with a multiplicity of infection (MOI) of 0.01. After 6 h of incubation (37 °C), the culture was centrifuged, treated with chloroform (10% v/v) and filtered (0.2 µm). Phage concentration was assessed by plaque counting (PFU mL^−1^) and phage stock was stored at 4 °C.

### Phage characterization

#### Transmission electron microscopy analysis

Phage particles (> 1 × 10^8^ PFU mL^−1^) were collected by centrifugation (1 h, 25 000 × *g*, 4 °C) and washed twice with water. Then, phages were deposited on copper grids with a carbon coated Formvar film grid and stained with 2% (w/v) uranyl acetate (pH 4.0). The visualization was performed on a transmission electron microscope (TEM), a Jeol JEM 1400 (Tokyo, Japan).

#### DNA isolation, genome sequencing, annotation, and comparative analysis

FJ1 genomic DNA was isolated using the phenol–chloroform-isoamyl alcohol method as previously described [26]. The DNA samples were used for the construction of the whole genomic library using TruSeq® Nano DNA Library Prep Kit. DNA fragments were sequenced in Illumina MiSeq, using 300 bp paired-end sequencing reads. After removing low quality bases, reads were de novo assembled using Geneious Prime. The assembled genomes were scanned through MyRAST for open reading frames [27] and tRNAscan-SE for tRNAs [28]. Protein functions were searched using BLASTP against NCBI nonredundant protein database and using HHpred against Protein Data Bank database, in all using an E-value 1 × 10^−5^ as threshold.

#### Lytic spectra determination and efficiency of plaquing

The host range was evaluated against a panel of 63 well-characterized ETEC strains provided by the LREC of the University of Santiago de Compostela (USC), Spain [3]. The host recognition rate was assessed by phage spot test: 10 µL FJ1 (1 × 10^8^ PFU mL^−1^) were placed onto each bacterial lawn prepared over LB agar and containing the respective mid-log grown strain suspended in LB with 0.6% (w/v) agar. After incubation (37 °C), plates were checked for clear zones. The efficiency of plaquing (EOP) was then performed in sensitive strains, spotting phage serial dilutions (starting from 1 × 10^8^ PFU mL^−1^) onto the respective bacterial lawns. The relative EOP was calculated by dividing the titre (PFU mL^−1^) of each susceptible strain by the titre of the relevant propagating host and was scored as follows: 0 (no lysis), 1 (≤ 50%), 2 (> 50%—100%), 3 (> 100%) and lysis from without (LFW).

#### One-step growth curve

An O/N-grown culture of HFJ1 was 100-fold diluted in 20 mL LB and incubated until reaching an OD_600nm_ of 0.3. Resultant cultures were then centrifuged (7000 × *g*, 5 min, 4 °C), resuspended in 5 mL fresh LB medium, and mixed with 5 mL FJ1 suspension to reach a MOI of 0.001. A subsequent incubation (37 °C for 10 min) allowed phage adsorption to bacterial cells. After centrifugation (7000 × *g*, 5 min, 4 °C), the pellet was resuspended in 10 mL LB broth. Samples were taken every 5 or 10 min, for 35 min, and plated immediately. The one-step growth curve was then analysed.

#### Identification of the phage receptor class

The type of phage receptors (carbohydrate or protein-based) on the bacterial surface was identified following the protocol proposed by Kiljunen et al. [29]. Phage host cultures were treated with: (1) sodium acetate (50 mM, pH 5.2) (control); (2) sodium acetate containing 100 mM periodate at room temperature for 2 h (to inactivate carbohydrates); or (3) proteinase K (0.2 mg mL^−1^) at 37 °C for 3 h (to inactive outer membrane proteins). Afterward, the phage was incubated with the treated host cells for 5 min at 37 °C, and the adsorption was measured by plaque counting (PFU mL^−1^) after serial diluting in SM buffer (5.8 g L^−1^ NaCl (PanReac); 2 g L^−1^ MgSO_4_·7H_2_O (VWR); 50 mL L^−1^ 1 M Tris–HCl pH 7.5 (VWR)). The phage adsorption rate (%) was obtained by subtracting the concentration of phage non-adsorbed to the initially present, and then divided by the initial phage titre. Each assay was performed at least 3 times.

#### Microencapsulation effectiveness on protecting/releasing phages from/in simulated gastrointestinal fluids

A previously optimized alginate/CaCO_3_ encapsulation method [20] performed by the Molecular Microbiology Group of the Autonomous University of Barcelona (Spain) was used to obtain FJ1 capsules. FJ1 released from the provided microcapsules was evaluated in both simulated gastric fluid (SGF, pH 3.0) containing 0.85% (w/v) NaCl and 3 mg mL^−1^ pepsin (Sigma-Aldrich) and simulated intestinal fluid (SIF, pH 6.5) with 1 mg mL^−1^ pancreatin (Sigma-Aldrich) and 10 mM bile salts (Sigma-Aldrich) [20]. After immersion, the suspensions were incubated at 37 °C and 120 rpm (ES-20/60, Biosan). Samples were taken at 0 min, 30 min, and 60 min, and phage titration was performed based on Colom et al. [30].

To evaluate the efficiency of the capsules in the protection or release of phages in both fluids, the concentration (PFU mL^−1^) of encapsulated phages during contact with them was evaluated: encapsulated phage (EF) = total phage concentration (C_t__otal_) - concentration of non-encapsulated phages (C_free_)_._ C_total_ was obtained by plating the whole suspension of wet microcapsules in LB with 0.6% (w/v) agar (the agar gelation will degrade microcapsules by sequestrating divalent ions important for the stability of the alginate matrix). C_free_ was attained after filtering the encapsulated suspension through a 0.2 μm PES filter (Frisenette) (to retain capsules) and titrating the filtered phage.

#### FJ1 activity against ETEC infected IPEC-1 cultured cells

The neonatal intestinal porcine cell line IPEC-1 (CVCL_2245) purchased from DSMZ-German Collection of Microorganisms and Cell Cultures GmbH was used for cell culturing. Cells were maintained in Dulbecco’s Modified Eagle’s Medium (DMEM, Biochrom) and Ham’s F-12 (Biochrom) (1:1) supplemented with 10% (v/v) fetal bovine serum (FBS, Biochrom) and 1 × ZellShield (Biochrom), at 37 °C in a humidified atmosphere with 5% CO_2_ (HERAcell 150). IPEC-1 cells were subcultured following the manufacturer’s recommendation (seed out at ~ 10^6^ cells/80 cm^2^ flask; split almost confluent culture 1:4 once or twice a week using trypsin/EDTA for about 10 min). Cells used in this study were subcultured with passages from 12 to 26.

The infection model of EC43, an ETEC O9:H9, on IPEC-1 cells and the ability of FJ1 to control the infection was firstly optimized with free phage, in 96-well plates (0.32 cm^2^ area) and then evaluated on 12-well plates (3.85 cm^2^ area). Accordingly, FJ1 antimicrobial efficacy was evaluated in IPEC-1 cultured cells, against ETEC O9:H9. Briefly, 0.1 mL of 5 × 10^5^ cells mL^−1^ were seeded in 96-well plates, washed with 10 mM PBS after 24 h, and infected with an O/N grown culture (~1 × 10^7^ CFU mL^−1^) suspended in DMEM/Ham's F-12 with 10% FBS (assay medium). Wells without infection were kept as control. After a 2 h incubation at 37 °C and 5% CO_2_, infected cells were washed with 10 mM PBS, treated with FJ1 (~1 × 10^6^ PFU mL^−1^) suspended in assay medium and incubated with agitation at 120 rpm. To calculate the MOI, CFU count was recorded at time zero of the phage infection: the incubation with 30% of the mixture 1:250 trypsin with EDTA (0.25%/0.02%) (Biochrom) (37 °C, 5% CO_2_, 15 min) allows bacterial release from enteric cells and dislodge the IPEC-1 cells from the plastic surface. Serial dilutions in 0.9% NaCl (w/v) were done using 5 mM ferrous ammonium sulfate (FAS), a virucide that will avoid CFU underestimation. Bacterial counts then were monitored at 2 h, 4 h, 6 h, and 24 h. The same infection model was then used in cells cultured in 12-well plates (starting from the same concentration of cells). Thus, 24 h after confirming cell confluence, they were washed and infected as before. The medium used herein was used as a control. Now, free or encapsulated phage resuspended in assay medium (pH adjusted to 6.5 to mimic intestinal conditions) were tested, in the same concentration, ~1 × 10^6^ PFU mL^−1^. The MOI was assessed as before. CFUs were also quantified 3 h, 6 h, and 24 h after phage administration. The adhesion of ETEC to epithelial cells was achieved without agitation, but phage treatment was performed using 60 rpm.

The effect of the encapsulated phage in ETEC-infected IPEC-1 cultured cells was visualized using an inverted microscope (DMI 3000B, Leica), after 6 h and 16 h. For that, and as described above, IPEC-1 cells were seeded in 24-well plates (better fitting the microscopic field), washed, ETEC-infected and phage-treated (with microcapsules containing ~1 × 10^6^ PFU mL^−1^). Cells were washed with PBS before observation.

#### Bacteriophage insensitive mutants—evaluation of phenotypic costs

For inducing the formation of BIMs (BIMFJ1), FJ1 was inoculated into a mid-log phase grown culture of EC43 (MOI 0.01) and incubated for 24 h with shaking (120 rpm). The resulting culture was then plated in LB agar and incubated O/N. Afterward, 10 colonies were picked and streaked at least three times into new plates to guarantee phage purity. The absence of phage infection was confirmed by plating serial dilutions of FJ1 on the lawns of BIMs (EOP).

The vulnerability of ten BIMFJ1 (BIM1.1 – BIM1.10) to the pig complement system was firstly assessed using serum, obtained by centrifugation of pig blood. The wild-type (WT) EC43 strain and BIMFJ1mid-log phase cultures (OD_600nm_ = 0.3) were diluted to obtain a 5 × 10^5^ CFU mL^−1^, mixed with porcine serum (3:1 (v/v)), and incubated for 1 h at 37 °C. The concentration of viable bacterial cells remaining in the suspension was obtained through CFU counting. Heat-inactivated serum (56 °C for 30 min) was used as a negative control.

Five of the previously tested BIMs (1.1, 1.4, 1.6, 1.9, 1.10) were further evaluated for their ability to adhere to swine intestinal cells compared to the originating strain (WT EC43). IPEC-1 cells were cultured in 96-well plates and infected as described above. The bacterial count was assessed after 2 h (CFU cm^−2^).

Finally, the other 3 selected BIMFJ1 (according to the phenotypes observed in the previous assays (1.1, 1.6 and 1.10) and WT EC43 were analysed considering the capability to cause death and to depreciate the health index in a *Galleria mellonella* model. Larval survival experiments were adapted from previous studies with small changes [31]. The larvae were raised in the dark at 25 °C with a pollen grain and bee wax diet. Worms of the final instar stage, with approximately 250 mg, were selected for the following experiments as previously reported [32]. Concisely, overnight cultures of each strain were harvested, washed, and adjusted to a final concentration of 2 × 10^6^ CFU mL^−1^ in PBS. The cell suspensions were normalised using optical density (OD_600_) and the CFU mL^−1^ were confirmed by the culture plate counting method. Each larva (*n* = 10 × 5 groups) was injected with 5 µL of bacteria (using a microsyringe adapted in a micrometre) into the hemolymph, via the hindmost left proleg, previously disinfected with ethanol 70% (v/v). Larvae from the negative control group were injected with 5 µL of PBS. After injections, larvae were placed on Petri dishes and remained in the dark at 37 °C for 72 h. The mobility of the larvae was assessed daily. If no movement was observed, they were considered dead. Additionally, the health index of the *G. mellonella* larvae was determined based on four indicators: larvae activity, cocoon formation, melanisation and survival. All experiments were repeated at least three times on separate days.

### Statistical analysis

The statistical analysis of the results was performed using GraphPad Prism 6. Results were compared using t-test, One-way ANOVA or Two-Way ANOVA. Kaplan–Meier survival curves were plotted, and statistical differences were determined by log-rank Mantel-Cox statistical test. All tests were performed with a confidence level of 95%. Differences were considered statistically different if *p*-value ≤ 0.05.

## Results

### Phage characterisation

**Figure 1:**
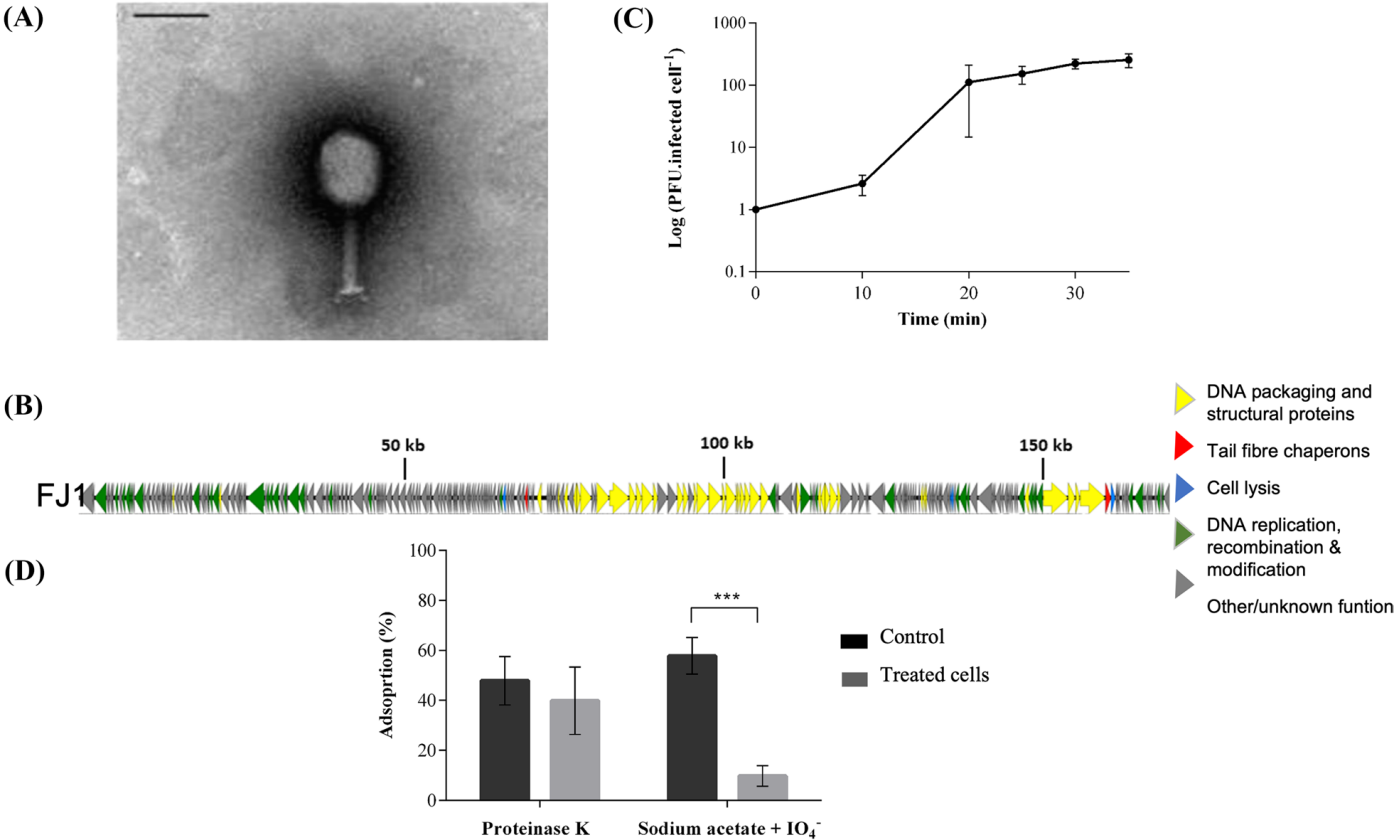
**Phage characterization. A** Microscopy observation. Transmission electron micrograph of phage FJ1. Black scale bar represents 100 nm; **B** One-step growth curve of FJ1 on the host strain, HJ1 (PFU per infected cell). Error bars represent standard deviation for an average of three experiments; **C** Genomic analysis. Representation of FJ1 genome: coloured arrows indicate open reading frames according to the putative function. Image was created using EasyFig; **D** Investigation of phage receptors. Effect of proteinase K (0.2 mg mL^−1^) and periodate (50 mM sodium acetate, pH 5.2, 100 mM IO_4_^−^) on phage adsorption (%) to host cells. Control assays were performed using distilled water. Errors bars represent standard deviation for an average of three experiments. Statistical significance of differences between treated and untreated groups was determined using the *t* test (* *p*-value < 0.05; *** *p*-value < 0.001; **** *p*-value < 0.001).

TEM images indicated that FJ1 is as tailed phage (*Caudovirales* order), with typical traits of the *Myoviridae* family members. The phage head was 82 nm × 64 nm and the tail length was 114 nm (Figure 1A). The phage genome, deposited in the GenBank with the accession number MZ170040.1 is a linear dsDNA molecule of 170.1 Kb (268 coding sequences), with a GC content of 35.4% and nine tRNA genes (Figure 1B). Core phage proteins have been identified, such as structural proteins related to DNA packing, DNA replication, DNA recombination and modification and cell lysis. Nevertheless, more than 50% of the proteome has no assigned function. No integration genes or genes potentially toxic to other bacteria were found.

The lytic spectrum of FJ1 against 63 ETEC strains of different serotypes is detailed in Additional file 1. There it can be observed that only one strain, EC43, was able to propagate the phage. However, despite the narrow spectra, FJ1 scored high efficiency of propagation (EOP > 100%), becoming a strong candidate for the establishment of the proof-of-concept of phage antimicrobial efficiency in ETEC-infected cultured cells.

The fitness parameters of this phage were evaluated by one-step growth curve (Figure 1C). FJ1 displayed short latency periods—10 min—and produced 96 phages per infected cell.

### Phage receptors

When EC43 was treated with periodate, the adsorption rate of FJ1 significantly decreased (*p*-value < 0.001) by 48.0% ± 10.4% compared to untreated cells. Phage adsorption was not affected when cells were treated with proteinase K (Figure 1D).

### Phage sensitivity to pH and release in the gastrointestinal fluids

**Figure 2:**
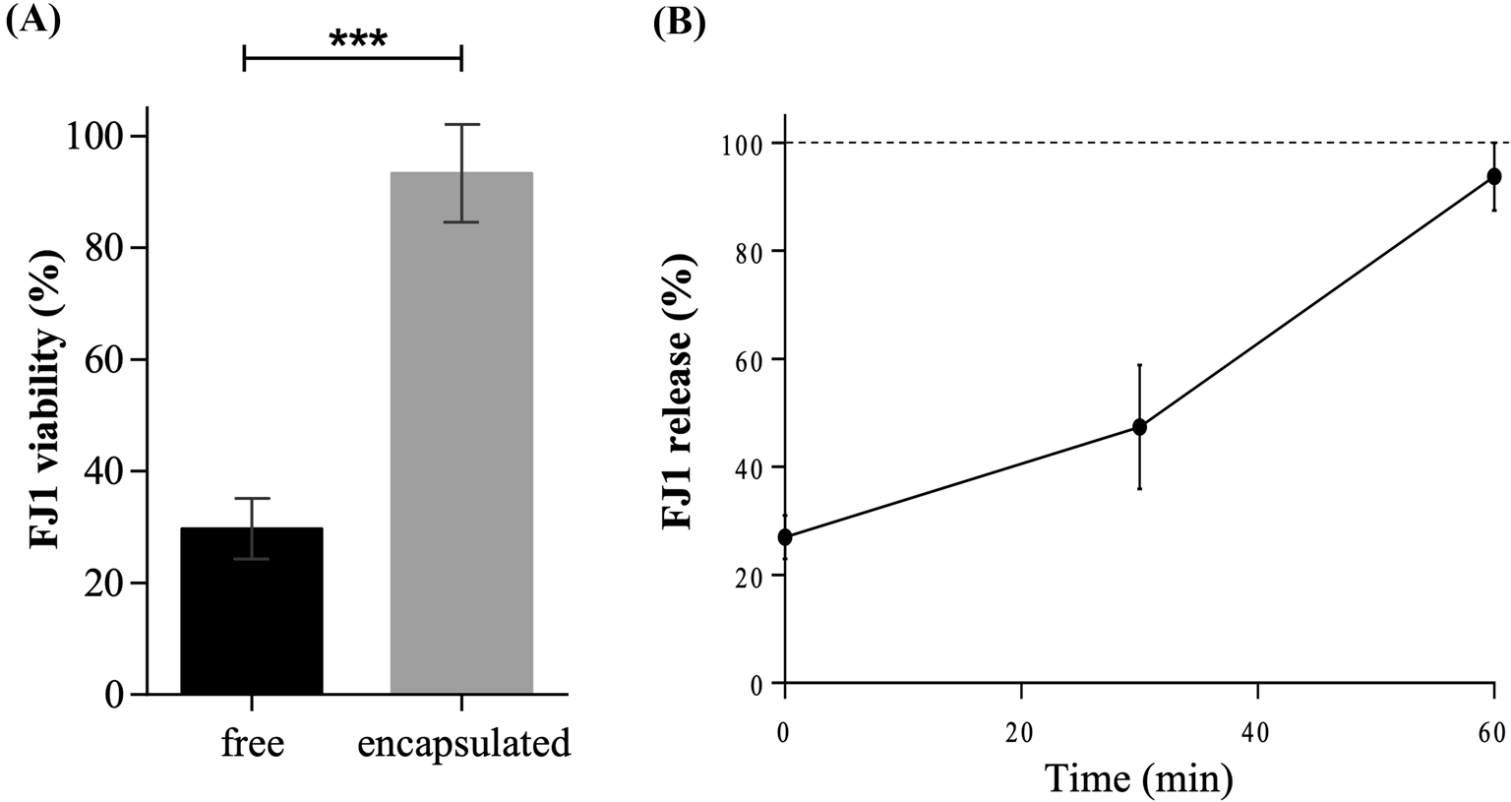
**Phage encapsulation. A** FJ1viability in SGF. Effect of the gastric fluid (pH 3.0) in non-encapsulated and encapsulated phage 1 h after incubation at 37 °C. Error bars represent standard deviation for three experiments. Statistical significance of differences between free and encapsulated groups was determined using the* t* test (*** *p*-value < 0.001); **B** Release of alginate/CaCO3 encapsulated FJ1 in SIF. Percentage of phage release in the intestinal fluid (pH 6.5). The phage was quantified 0 min, 30 min and 60 min after incubation at 37 °C, and compared with the total phage provided in the capsules (Phage release (%) = C_time point_/C_total_)*100). Errors bars represent standard deviation for at least three experiments.

After 1 h of immersion in SGF only 29.7% ± 5.4% of the free FJ1 particles were viable. However, when encapsulated in CaCO_3_/alginate, 93.3% ± 8.7% remained viable and within the capsules (*p*-value < 0.001) (Figure 2A). In SIF, 27.0% ± 4.0%, 47.4% ± 11.5% and 93.7% ± 6.3% of the FJ1 particles were progressively released, respectively at 0 min, 30 min and 60 min (Figure 2B).

### FJ1 antimicrobial activity in an IPEC-1 infection model

**Figure 3:**
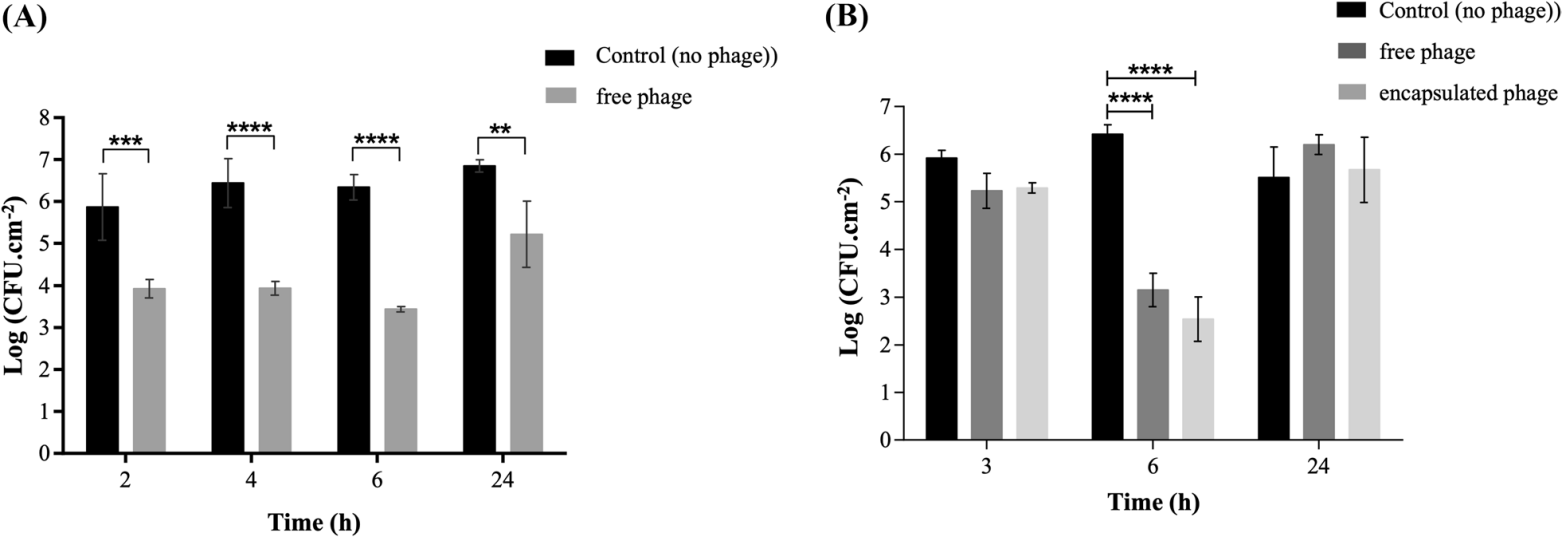
**Activity of phage FJ1 against ETEC EC43 on IPEC-1 cultured cells. A** Free phage: the effect of FJ1 towards an ETEC O9:H9 cells, adhered to an IPEC-1 monolayer (cultured for 24 h in 96-well plates) was assessed. Bacterial load (CFU cm^−2^) was measured at 2 h, 4 h, 6 h and 24 h. Errors bars represent standard deviation for three experiments. Statistical significance of differences between treated and untreated groups was calculated using a two-way ANOVA test (** *p*-value < 0.01; *** *p*-value < 0.001; **** *p*-value < 0.0001); **B** Free and Microencapsulated phage: the effect of free and microencapsulated FJ1 towards ETEC O9:H9 adhered to an IPEC-1 cell monolayer (cultured for 24 h in 12-well plates, pH 6.5) was assessed and compared. Bacterial load (CFU cm^−2^) was measured at 3 h, 6 h and 24 h. Errors bars represent standard deviation for three experiments. Statistical significance of differences between treated and untreated groups was calculated using a two-way ANOVA test (**** *p*-value < 0.0001).

The antibacterial activity of FJ1 against EC43 (O9:H9), infecting IPEC-1 cultured cells in wells of 96-well plates, revealed that the phage (MOI = 3.5) was able to reduce the adhered cells on average, by 1.9 Log CFU cm^−2^, 2.5 Log CFU cm^−2^, 2.9 Log CFU cm^−2^ and 1.6 Log CFU cm^−2^ after 2 h, 4 h, 6 h and 24 h, respectively (Figure 3A). The initial concentration of EC43 adhered to intestinal cells was, in average, 6.0 ± 5.3 Log CFU cm^−2^. Assays with free and encapsulated FJ1, in wider layers of EC43-infected confluent cells (Figure 3B), showed that the maximum reduction of bacterial load occurred 6 h after treatment: both non-encapsulated and encapsulated phages were able to decrease bacterial content (6.4 ± 0.2 Log CFU cm^−2^ adhered to IPEC-1 cells), respectively by 3.3 Log CFU cm^−2^ (MOI = 9.8) and 3.9 Log CFU cm^−2^ (MOI = 11.2). No significant difference was observed between the antimicrobial performance of free and encapsulated phages (*p*-value > 0.05). At 3 h and 24 h, no significant differences were observed between treatments and control groups (*p*-values < 0.05).

### Microscopy analysis of pig cells

**Figure 4:**
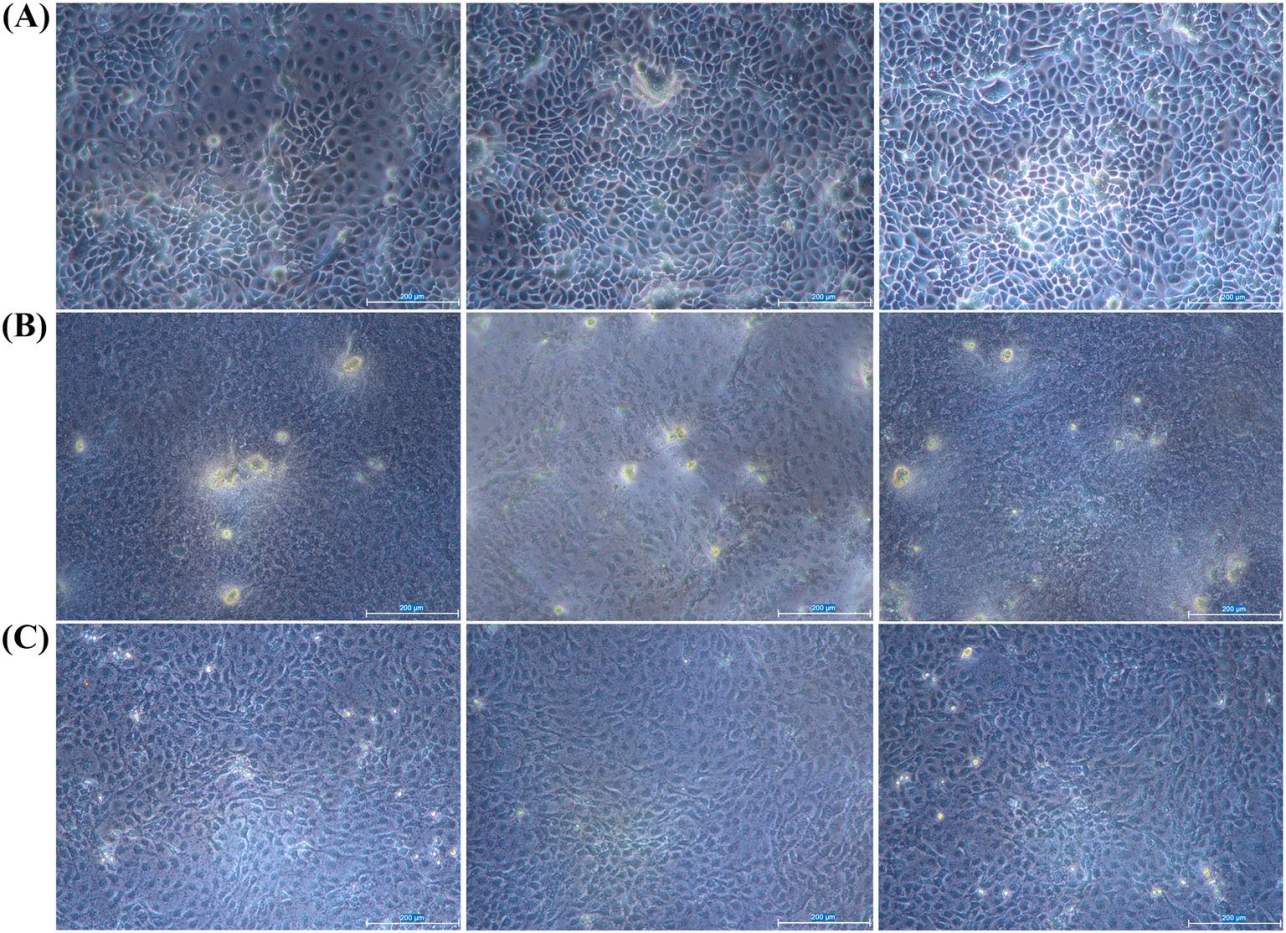
**Microscope images of cultured IPEC-1 cells. A** Typical morphology of IPEC-1 cells (control group); **B** IPEC-1 cells infected with ETEC O9:H9; **C** IPEC-1 infected cells treated with phage. Images were obtained under bright field, 16 h after phage administration. White bar: 200 µm.

When visualized microscopically after 6 h, no visual differences were recorded between the morphology of non-infected and ETEC-infected IPEC-1 cell monolayers. In the subsequent observation performed 16 h after co-culture, the cells of the infected group already revealed morphological alterations, becoming shrunken and without regular borders, comparatively to the control. The cell integrity of the control was confirmed by their typical morphology, with consistent size and sharp edges. An intermediate status seemed to be promoted by phage-treated cells, with more defined borders and typical size (Figure 4).

### Assessment of BIMFJ1 phenotypic costs

**Figure 5:**
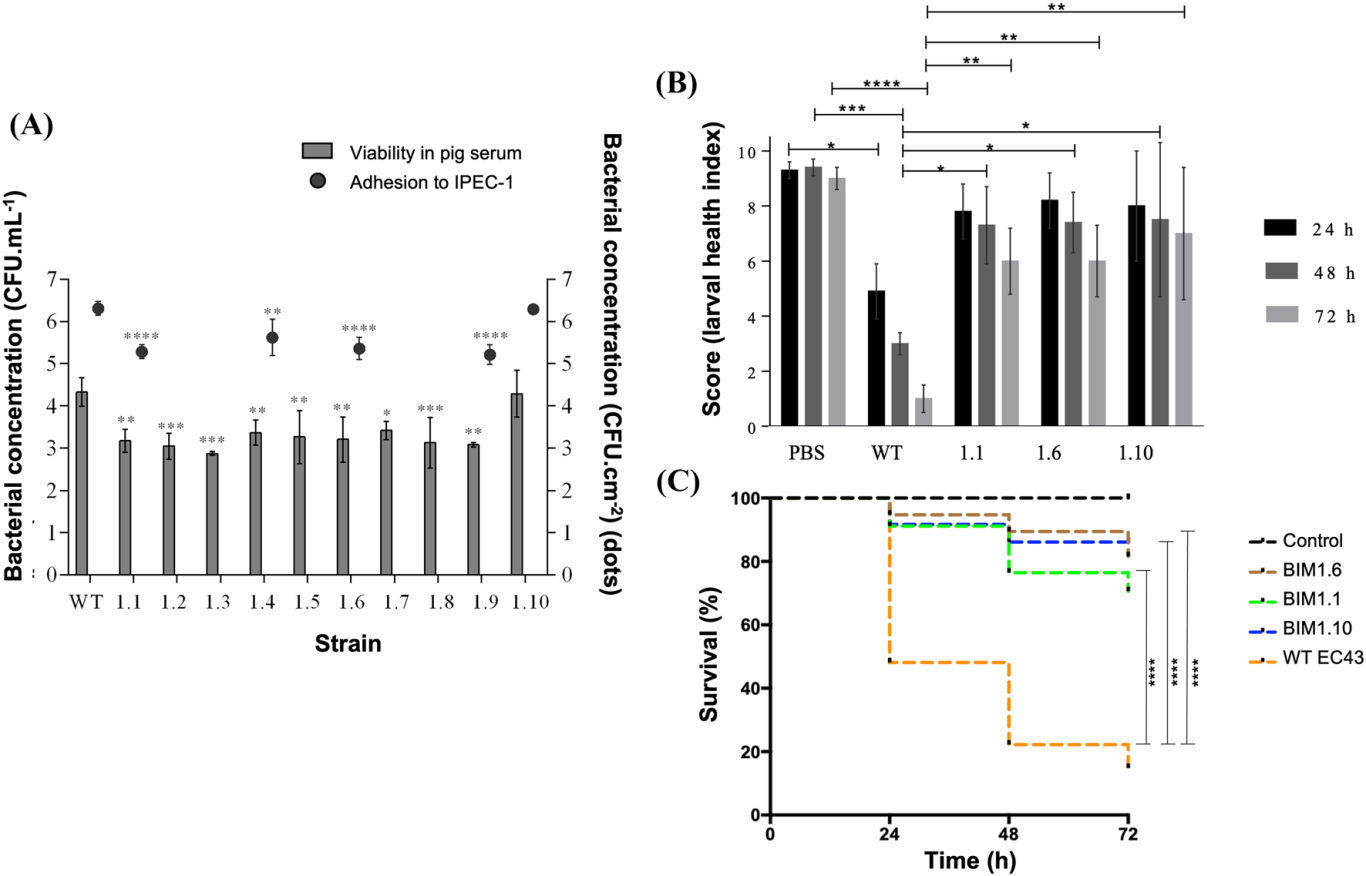
**Assessment of BIMFJ1 phenotypic costs.** **A** Bacterial viability in pig serum and adhesion to IPEC-1 cultured cells. Bars indicate the concentration (CFU mL^−1^) of each strain (BIM 1.1, 1.2, 1.3, 1.4, 1.5, 1.6, 1.7, 1.8, 1.9, 1.10 and EC43 WT) before and after incubation with pig serum. Dots represent the concentration (CFU cm^−2^) of each strain** (**BIM 1.1, 1.4, 1.6, 1.9, 1.10 and WT EC43 adhered to IPEC-1, 2 h after infection (MOI = 100). Errors bars represent standard deviation for an average of three repeated experiments. Statistical significance of differences between BIMs and the WT strain was determined using a one-way ANOVA test (* *p*-value < 0.05** *p*-value < 0.01; *** *p*-value < 0.001; **** *p*-value < 0.0001); **B** Health index in *G. mellonella* model. Larvae health index scores in wax moth larvae, 24 h, 48 h and 72 h post-infection with BIM 1.1, 1.6, 1.10 and EC43 and PBS (control). Statistical significances of differences between mutants, wild-type and control groups were calculated using a two-way ANOVA (* *p*-value < 0.05; ** *p*-value < 0.01; *** *p*-value < 0.001; **** *p*-value < 0.0001); **C** Survival rates of challenged *G. mellonella*. Larval survival rates, 24 h, 48 h and 72 h post-infection with BIM 1.1, 1.6, 1.10 and EC43 and PBS (control). All experiments were performed in triplicate and in a minimum of three independent assays. Statistical significance was calculated using the log-rank Mantel-Cox statistical test (**** *p*-value < 0.0001).

The performance of the complement system, part of the host’s innate immune response, promoting, for example, bacterial lysis, was analysed using pig serum. After confirmation of BIMFJ1 phenotype in 10 mutant strains (1.1, 1.2, 1.3, 1.4, 1.5, 1.6, 1.7, 1.8, 1.9, and 1.10), by observing the absence of phage infection, the challenge with serum led to conclude that overall, 90% of them suffered a reduction of, in average, 1.2 ± 0.2 Log CFU mL^−1^ comparatively to WT EC43 (*p*-value < 0.01) (Figure 5A). Only mutant 1.10 seemed not to be significantly susceptible to serum activity when compared with the originating strain (*p-*value > 0.05). The control tests performed with the inactivated serum did not influence the bacterial load concentration (data not shown), confirming that the reduction observed was only due to the bactericidal action of the serum. Figure 5A also illustrates the results of cell-adhesion assays. Almost all BIMs—1.1, 1.4, 1.6, and 1.9 demonstrated a reduced cell-adhesion ability (on average 5.4 ± 0.2 Log CFU cm^−2^) compared to the parental strain (6.3 ± 0.2 Log CFU cm^−2^) (*p*-value < 0.05). Again, no difference was observed between BIM 1.10 and WT EC43 (*p*-value > 0.05). With regards to the health index of *G. mellonella* evaluated throughout 72 h after challenging with WT EC43 and BIMs 1.1, 1.6, and 1.10, it was observed that groups of larvae receiving mutant strains had higher health index scores at 48 h and 72 h post-injections (Figure 5B), displaying increased movement, cocoon formation, and slightly less melanisation (overall average score: ≥ 7.3 vs 3.0, after 48 h (*p*-value < 0.05) and ≥ 6.7 vs 1.8, after 72 h (*p*-value < 0.01)). Furthermore, compared to the negative control (larvae only injected with PBS), all BIMs had statistically similar results (*p* > 0.05). Accordingly, the injection of WT EC43 allowed only 48.1%, 22.2% and 14.8% of the larvae survival, respectively after 24 h, 48 h and 72 h, while more than 91% of the BIMs-injected larvae were still alive 24 h after challenge and more than 70% after 48 h and 72 h (*p*-value < 0.0001) (Figure 5C).

## Discussion

Nowadays, the swine industry is facing a huge challenge due to the strict restrictions on the prescription of conventional antimicrobials for clinical purposes. The use of phages to tackle enteric diseases in young pigs seems to be an important option to address such challenges. In the context of ETEC-induced neonatal diarrhoea, the way phage therapy can relate to traditional sow vaccination should be elucidated. With the aim of protecting the animal against this pathology, both approaches can potentially complement each other in their principle of action. The vaccination of sows will enable the transfer of antibodies against the most common ETEC serotypes, through colostrum. However, as ETEC are non-invasive to cells, only antibodies that reach the small intestine of piglets will bind to bacterial adhesins inhibiting bacterial adhesion to the enterocytes. These will be the important players in providing immunity against the disease [8]. The administration of phages will in turn specifically destroy bacteria adhered to the intestinal mucosa releasing new progeny in exponential proportions. By reducing local bacterial load, phages will make the action of the immune system more efficient and pragmatic.

It is now known that oral intake of phages may decrease their viability due to adverse conditions of the gastrointestinal tract, and therefore, the protection of the phage can increase the concentration of viable particles that reach the intestinal mucosa upstream. In this work, an exclusive virulent phage, FJ1, has been fully characterized and used to establish a proof-of-concept concerning its ability to reduce the ETEC load adhered to cultured intestinal cells. The challenging strain, EC43, is a common “prototype” of an isolate implicated in neonatal porcine diarrhoea due to its serotype O9:H9 and virulence traits (fimbriae (F5, F41)) [3]. This supports the use of the neonatal cell line, IPEC-1. The impact of phage-resistant mutants’ emergence among bacteria on therapeutic effectiveness was further analysed.

The characterization of FJ1 revealed that it is a safe phage for therapeutical purposes, with no integrases or toxic genes that can be transferred by transduction to neighbouring bacteria. Despite the very narrow lytic spectra against a selection of ETEC strains belonging to the most frequently identified serotypes implicated in porcine colibacillosis worldwide, its high efficiency on propagation in EC43 (EOP > 100%) supported the selection of FJ1 as a proof-of-concept model in cultured cells from the piglet intestine.

The advantage of CaCO_3_/alginate microcapsules in protecting phage against simulated severe conditions of the gastric environment was already reported [20]. Based on the results (Figure 2) it can be inferred that approximately 73% of the viable phage might reach the intestine if protected, and once there, an immediate availability of ~27% of infectious viral particles occurs, before the almost full release, that occurred 1 h after. While phage release at low pH (SGF) was prevented by the alginate retreatment due to the protonation of the carboxyl groups, it was promoted by alginate swelling, occurring at higher pH (SIF) [33, 34]. When assessing the viability of phages in SGF, the pH of 3 was selected to simulate the most severe pH conditions that they may face in the stomach of weaning pigs. In the case of suckling piglets, they have a higher gastric pH (5 to 6) due to the buffering actions of colostrum and milk. If phage release occurs early in the stomach, it is highly likely that, in general, phages will tolerate those favourable conditions [35]. The lower feed retention time in the stomach of piglets promoted by the liquid diet will also decrease any deleterious effects.

Promising results were observed in IPEC-1 monolayers of 0.32 cm^2^ area, infected with ETEC cells and treated with free FJ1 (maximum reduction of 2.9 Log CFU cm^−2^ after 6 h), encouraging, thus, further assays with microencapsulated phage, in wider IPEC-1 monolayers. Under the same pH as that of the piglet intestine, successful indicators were obtained: the microencapsulated FJ1 decreased EC43 load in 3.9 Log CFU cm^−2^ (> 99.9%), also 6 h after phage administration (Figure 3). Phage effectiveness in decreasing bacterial contamination in intestinal cell lines was previously observed by Mirzaei et al. [36] in HT-29 and Caco-2, with reductions of 1.0 to 4.0 Log CFU mL^−1^ of ESBL *E. coli* strains for 8 h. More recently, Kim et al. [37], reported that the phage EK99P-1 was able to alleviate ETEC K99-induced barrier dysfunction and inflammatory response of IPEC-J2 cultured cells. In this study, the protective effect, that FJ1 seems to have on EC43-infected cells (at least 16 h after phage administration), was observed microscopically, showing an apparent delay in cell damage. As expected, based on previous reports [38, 39], there was a decrease in FJ1 antibacterial activity between 6 and 24 h after administration (Figure 3B). The emergence of bacteriophage-resistant strains, BIMs, (confirmed by phage spot test against recovered colonies) may have contributed to biomass regrowth [40]. However, previous knowledge also anticipated that newly emergent phage-resistant phenotypes would bring important fitness costs for mutant strains [41], and the reduction of virulence was, in fact, confirmed here. This is, therefore, a good indicator encouraging the applicability of phages to treat ETEC infections, however, further in vivo experiments are crucial to support this novel approach. It has actually been reported that BIMs may acquire phage resistance by several means, commonly through mutations in phage receptor sites, interfering with phage attachment [42]. After taking note that the FJ1 host receptor was at the level of the LPS structure, the absence of phage adsorption to BIMs indicated that host mutations occurred in the LPS. Similar to what was previously observed [43], this study also suggests that changes in LPS complex influence the action of the immune system. For example, nine out of ten BIMs tested in the serum suffered a decrease in concentration when compared to the WT strain (Figure 5A). It is already known that the length of the O-antigen chain prevents the antibody-mediated deposition of complement components at the bacterial cell surface. As a result, the O-antigen structure protects bacteria from lysis by complement [6]. Additionally, possession of an O-antigen has been demonstrated to enable the evasion of pathogens due to innate resistance mechanisms involving complement activation. This is the case of phagocytosis carried out by macrophages [44]. This seems to be in line with the idea that mutations may have caused the immunostimulant lipid A region to be more exposed to the action of the proteins of the complement system. It is known that one of the *E. coli* mechanisms to overcome complement activation, is by synthesizing an LPS of low immunogenicity [7]. Differences in immune system response to BIMs were further supported by subsequent experiments in *G. mellonella* larvae. None of the mutant strains tested (BIM 1.1, 1.6 or 1.10) caused disease (Figure 5B), at least within 72 h, and the health index of larvae remained statistically similar to that of the group without infection (*p*-value > 0.05). This suggested that the larval innate immune system was able to detect and target such strains with increased immunogenicity. It is important to mention that *G. mellonella* in vivo model has been used to study *E. coli* pathogenicity. Based on this model, it is possible to evaluate the immune innate response of *G. mellonella* against bacteria, namely, melanization and antimicrobial peptides (AP) production (such as cecropin and gloverin) [45]. To achieve a more close experimental design to the porcine model, pig serum has been also studied, since it contains a variety of AP, inhibiting bacterial cell growth and constituting a host's innate immune system against bacterial infection [46]. AP disrupt the membrane of bacterial cells, and the lack of protection of the LPS barrier leads AP to target the bacterial membrane. Curiously, BIM10.0 seemed to have escaped from inactivation in the pig serum, but not from the larval immune system. Mutations at the LPS level might not have supported the accessibility of the complement proteins but still allowed cells to be targeted by larval AP. For example, also in BIMs described for *Salmonella* phages, a different range of mutations was observed, suggesting the occurrence of diverse mechanisms of cell modification associated with resistance to phages [19]. Even though the adhesion-associated proteins (e.g., flagella, fimbriae) may not be different between BIMs and WT EC43, since these were not the sites of phage adsorption, a lower IPEC-1 colonization ability (almost 1 Log CFU cm^−2^ in average) was observed for the BIMs more susceptible to pig serum comparatively to the parental strain (*p*-value > 0.05) (Figure 5A). Possibly, ETEC strains modified at the level of the LPS inner core decreased their effectiveness on intestinal colonization, as observed by Kuo et al. [46] for EHEC, reporting lower ability to colonize the intestine of *Caenorhabditis elegans* and mice. Such modifications at the cell wall level may also have decreased adhesion due to a higher susceptibility to host defence proteins secreted by gut epithelial cells upon colonization with ETEC. This lack of effectiveness in colonizing the intestinal cells seems of relevant importance since this is the first step of the infection by diarrhoeagenic pathotypes such as ETEC.

Overall, the results of this study suggest the success of the use of phages to control severe diarrhoea caused by *E. coli* in young pigs. Although FJ1 has not been selected by its spectrum, but rather to provide proof-of-concept of its antibacterial effectiveness, such a narrow range may raise a pressing question: “what impact other low-spectrum phages will have on the feasibility of using phage therapy to treat ETEC-induced disease?”. Several researchers propose to address these gaps by using phage cocktails to extend their scope of action against infections [47]. It is important that the development of a particular phage cocktail is dynamic and includes the possibility of reformulation under ongoing drug approval. For example, the so-called “magistral preparations”, a personalized approach to using cocktails “à la carte”, should be prepared to become available after a simple EOP test assessing the susceptibility profile of the isolated agent. Another important fact was to verify that the appearance of phage-resistant bacteria sometime after the administration may be circumventable. If on the one hand, one can count on the fitness cost of such a new phenotype, supporting the action of the animal immune system, on the other hand, the use of cocktails may contribute to overcoming a possible loss of efficiency. The design of mixtures including cross-infecting phage types (with different receptors), phages isolated from phage-resistant hosts, or synthetic phages recognizing different receptors are some examples that are being pointed out by many researchers [47]. An expeditious way to administer such a product, in the form of microcapsule suspensions, would be *per os*, with the aid of a syringe, for example, ensuring that each animal receives the desired number of phages.

In summary, phage FJ1 revealed to be a useful tool to control ETEC adhered to intestinal cells, and according to the results, it has a high potential to succeed in treating pig enteric infections. The advantages of administering the phage inside pH-responsive microcapsules were evident, foreseeing phage oral administration. More phages, with different host receptors shall be isolated and characterized in subsequent works, to comprise a cocktail to be tested in vivo. In addition, it seems central to realize whether the low spectrum of phage lysis against most ETEC strains is a common standard. If so, both the investigation of what mechanisms are conferring immunity to the host and what strategies phage are using (or can use) to counteract such mechanisms, will be valuable.

## Supplementary Information

**Additional file 1**. **Lytic spectrum and EOP of phage FJ1 against 63 ETEC strains** [3, 10]. The EOP was scored as follows: 0 (no lysis), 1 (≤ 50%), 2 (> 50%—100%) and 3 (> 100%). LFI: for lysis from within; LFW: lysis from without. NA: Not assigned, ONT: O non-typeable, HNM: H non motile.

## Data Availability

The datasets used and/or analysed during the current study are available at Science DB databank (https://www.scidb.cn). https://doi.org/10.57760/sciencedb.06665.

## Abbreviations

ETEC: enterotoxigenic *E. coli*
LPS: lipopolysaccharides
Phages: bacteriophages
BIMs: bacteriophage-insensitive mutants
FJ1: VB_EcoM_FJ1
LB: lysogeny Broth
MOI: multiplicity of infection
STEC: Shiga toxin-producing *E. coli*
EOP: efficiency of plating
LFW: lysis from without
SGF: simulated gastric fluid
SIF: simulated intestinal fluid
EF: encapsulated phage
DMEM: Dulbecco’s Modified Eagle’s Medium
FBS: fetal bovine serum
FAS: ferrous ammonium sulfate
WT: wild type
AP: antimicrobial peptides
