# Supplementary material for: Effect of phage vB_EcoM_FJ1 on the reduction of ETEC O9:H9 infection in a neonatal pig cell line
Source: Vet Res. 2023 Mar 22;54:26. doi: 10.1186/s13567-023-01157-x (PMC10035155; doi:10.1186/s13567-023-01157-x)
Supplement: Supplementary file 1 — Additional file 1. Lytic spectrum and EOP of phage FJ1 against 63 ETEC strains [3, 10]. The EOP was scored as follows: 0 (no lysis), 1 (≤ 50%), 2 (> 50%—100%) and 3 (> 100%). LFI: for lysis from within; LFW: lysis from without. NA: Not assigned, ONT: O non-typeable, HNM: H non motile. [file 13567_2023_1157_MOESM1_ESM.docx]

| **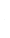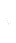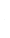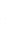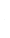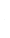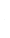Strain** | **Serogroup** | **Virulence profile** | **Origin** | **EOP_FJ1** |
| --- | --- | --- | --- | --- |
| EC2-Ph | NA:HNM | *Hly, LT, STb* | Post-weaning diarrhea | 0 |
| EC3-Ph | O45:H39 | *Hly, LT, F18* | No data | 0 |
| EC4-Ph | NA:HNT | *Hly, LT, STb, K88* | No data | 0 |
| EC5-Ph | O45:H39 | *Hly, LT, STb, K88* | No data | 0 |
| EC6-Ph | O108:H39 | *LT* | Post-weaning diarrhea | 0 |
| EC7-Ph | O45:H39 | *Hly, LT, F18* | Post-weaning diarrhea | 0 |
| EC8-Ph | O45:H39 | *Hly, LT, STb; K88* | Post-weaning diarrhea | 0 |
| EC9-Ph | O45:HNM | *Hly, STb, K88* | No data | 0 |
| EC10-Ph | O108:HNM | *Hly, LT, STb* | Post-weaning diarrhea | 0 |
| EC11-Ph | O108:HNM | *Hly, LT, Sta, STb, F18* | No data | 0 |
| EC12-Ph | O108:HNT | *Hly, Sta, STb, F18* | No data | 0 |
| EC13-Ph | O108:HNM | *Hly, LT, Sta, F18* | Post-weaning diarrhea | 0 |
| EC14-Ph | O108:HNM | *Hly, LT, Sta, F18* | Post-weaning diarrhea | 0 |
| EC15-Ph | O108:HNM | *Hly, LT, Sta, F18* | Post-weaning diarrhea | 0 |
| EC16-Ph | O108:HNM | *Hly, Sta, STb, F18* | No data | 0 |
| EC17-Ph | O108:HNM | *Hly, LT, Sta, STb, F18* | No data | 0 |
| EC18-Ph | O141:H4 | *Hly, Sta, STb, VT2e, F18* | Post-weaning diarrhea | 0 |
| EC19-Ph | O141:H4 | *Hly, Sta, STb, VT2e, F18* | Post-weaning diarrhea | 0 |
| EC20-Ph | O141:H4 | *Hly, Sta, STb, F18* | No data | 0 |
| EC21-Ph | O141:H4 | *Hly, Sta, STb, F18* | Post-weaning diarrhea | 0 |
| EC22-Ph | O141:H4 | *Hly, Sta, F18* | Post-weaning diarrhea | 0 |
| EC23-Ph | O157:HNM | *Hly, LT, Sta,STb, F18* | Post-weaning diarrhea | 0 |
| EC24-Ph | O157:HNM | *Hly, LT, Sta,STb, F18* | Post-weaning diarrhea | 0 |
| EC25-Ph | O157:HNM | *Hly, LT, Sta,STb, F18* | No data | 0 |
| EC26-Ph | O157:HNM | *Hly, LT, STb, K88* | Post-weaning diarrhea | 0 |
| EC27-Ph | O157:HNM | *Hly, LT, STb, K88* | Post-weaning diarrhea | 0 |
| EC28-Ph | O157:HNM | *LT, STb, K88* | No data | 0 |
| EC29-Ph | O157:HNM | *Hly, LT, STb, K88* | Neonatal diarrhea | 0 |
| EC31-Ph | O157:HNM | *Hly, LT, STb, K88* | Post-weaning diarrhea | 0 |
| EC33-Ph | NA:HNM | *Hly, LT, STb, K88* | Neonatal diarrhea | 0 |
| EC34-Ph | O7:H4 | *STb* | No data | 0 |
| EC35-Ph | O101:H9 | *Sta, K99/F41* | Post-weaning diarrhea | 0 |
| EC36-Ph | O45:H39 | *Hly, LT, STb, K88* | Post-weaning diarrhea | 0 |
| EC37-Ph | O101:H9 | *Sta, K99/F41* | Post-weaning diarrhea | 0 |
| EC38-Ph | O101:HNM | *Sta, K99/F41* | Neonatal diarrhea | 0 |
| EC39-Ph | O101:HNM | *Sta, K99/F41* | Neonatal diarrhea | 0 |
| EC40-Ph | O101:H10 | *Sta, K99* | No data | 0 |
| EC41-Ph | O101:H10 | *Sta, K99* | Neonatal diarrhea | 0 |
| EC42-Ph | O101:H10 | *Sta, K99* | Neonatal diarrhea | 0 |
| EC43-Ph | O9:H9 | *Sta, K99/F41* | Neonatal diarrhea | **3** |
| EC44-Ph | O182:H19 | *Sta, STb* | No data | 0 |
| EC45-Ph | O20:H9 | *Sta, P987* | Neonatal diarrhea | 0 |
| EC50-Ph | O138:H10 | *Hly, LT, STb, K88* | No data | 0 |
| EC51-Ph | O138:H10 | *Hly, LT, STb, K88* | Post-weaning diarrhea | 0 |
| EC52-Ph | O149:H10 | *Hly, LT, STb, K88* | No data | 0 |
| EC53-Ph | O149:H10 | *Hly, LT, STb* | Neonatal diarrhea | 0 |
| EC54-Ph | O149:H10 | *Hly, LT, STb, K88* | Post-weaning diarrhea | 0 |
| EC55-Ph | O149:H10 | *Hly, LT, STb, K88* | Neonatal diarrhea | 0 |
| EC56-Ph | O138:H14 | *Hly, Sta, STb, VT2e, F18* | Post-weaning diarrhea | 0 |
| EC57-Ph | O138:H14 | *Hly, Sta, STb, VT2e, F18* | Post-weaning diarrhea | 0 |
| EC58-Ph | O138:HNM | *Hly, Sta, STb, F18* | Post-weaning diarrhea | 0 |
| EC59-Ph | O138:HNM | *Hly, Sta, STb, VT2e, F18* | Post-weaning diarrhea | 0 |
| 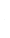EC60-Ph | O138:HNM | *Hly, Sta, VT2e, F18* | Post-weaning diarrhea | 0 |
| EC61-Ph | O138:H14 | *Hly, LT, Sta, STb, F18* | Post-weaning diarrhea | 0 |
| EC62-Ph | O138:H14 | *Hly, LT, Sta, STb, F18* | Post-weaning diarrhea | 0 |
| EC63-Ph | O157:H5 | *STb* | No data | 0 |
| EC64-Ph | O9:H9 | *Sta* | No data | 0 |
| EC65-Ph | O9:HNM | *Sta, STb, K88* | No data | 0 |
| EC66-Ph | O15:H45 | *STb* | No data | 0 |
| EC67-Ph | O45:H45 | *STb* | No data | 0 |
| EC68-Ph | O23:H16 | *Sta, STb, K88* | No data | 0 |
| EC69-Ph | O8:HNM | *STb* | No data | 0 |
| EC70-Ph | NA:HNM | *Sta, K99* | Neonatal diarrhea | LFW |
